# Vocal networks remain stable after a disturbance in Emei music frogs

**DOI:** 10.1002/ece3.5473

**Published:** 2019-07-23

**Authors:** Ke Deng, Jian-Guo Cui

**Affiliations:** ^1^ Chengdu Institute of Biology Chinese Academy of Sciences Chengdu China

**Keywords:** *Babina daunchina*, centrality, competitiveness, social network analysis, vocal network

## Abstract

Social network analysis has been widely used to investigate the dynamics of social interactions and the evolution of social complexity across a range of taxa. Anuran species are highly dependent on vocal communication in mate choice; however, these species have rarely been the subject of social network analysis. The present study used social network analysis to investigate whether vocal network structures are consistent in Emei music frog (*Babina daunchina*) after the introduction of a simulated exotic rival of varying competitiveness into the social group. We broadcasted six categories of artificial calls (either highly sexually attractive calls produced from inside male nests or calls of low sexual attractiveness produced outside nests with three, five or seven notes, respectively) to simulate an intruder with different levels of competitiveness. We then constructed vocal networks for two time periods (before and after the disturbance) and quantified three network metrics (strength, closeness, and betweenness) that measure different aspects of individual‐level position. We used the mean values of these network metrics to evaluate group‐level changes in network structure. We found that the mean strength, mean closeness and mean betweenness were consistent between two time periods in all ponds, despite the fact that the positions of some individuals had changed markedly after disturbance. In addition, there was no significant interaction effect between period and numbers of notes on the three network metrics. These finding suggest that the structure of vocal networks in Emei music frogs remain stable at the group level after a conspecific disturbance, regardless of the intruder's competitiveness.

## 1 INTRODUCTION

In recent years, social network analysis (SNA) has become a widely used framework in biology for investigating the links between individual behavior and group‐level patterns and processes (Croft, Darden, & Wey, 2016; Pinter‐Wollman et al., 2014; Sih, Hanser, & McHugh, 2009). The dynamic patterns of interactions among individuals generally vary based on ecological context such as climate, reproductive condition, and conspecific competition, which may result in temporal changes in group‐level network structure (Deng, Liu, & Wang, 2017; Patriquin, Leonard, Broders, & Garroway, 2010; Wey, Burger, Ebensperger, & Hayes, 2013). In turn, the dynamics of social networks commonly affect individual fitness (survival or reproductive success) and can also influence ecological and evolutionary processes at the population level (Croft, James, & Krause, 2008; Kurvers, Krause, Croft, Wilson, & Wolf, 2014; Montiglio, McGlothlin, & Farine, 2018; Wey, Blumstein, Shen, & Jordán, 2008). Although several studies have focused on temporal changes in social networks (Bar Ziv et al., 2016; Blaszczyk, 2017; Maldonado‐Chaparro, Hubbard, & Blumstein, 2015; VanderWaal, Atwill, Isbell, & McCowan, 2014), most studies have utilized association networks based on physical contact, spatial proximity, or co‐occurrence. To date, only a small number of studies have focused on vocal networks in animal populations (Fernandez, Vignal, & Soula, 2017; Kulahci, Ghazanfar, & Rubenstein, 2018; Kulahci, Rubenstein, & Ghazanfar, 2015; Snijders & Naguib, 2017).

Acoustic communication plays a crucial role in mate choice of most anurans. Generally, male anurans vocalize to attract mates and male calls provide pertinent information salient to females, including male body size, reproductive status and/or resources (Wells & Schwartz, 2007). Females assess the qualities of potential mates in large part by extracting the information in advertisement calls in order to make a choice. In addition, males are able to evaluate the competitiveness of rivals based on information in their advertisement calls, enabling them to adjust competitive strategies immediately, such as increasing call rate (Xu, Cui, Song, Brauth, & Tang, 2012), increasing the number of notes (Cui et al., 2016), or prolonging call duration (Botto & Castellano, 2016). For example, male serrate‐legged small treefrogs (*Kurixalus odontotarsus*) modulate the vocal complexity of their calls on the basis of the competitive context in real time, in order to maximize their competitiveness with minimal energy costs (Zhu et al., 2017). Thus, the vocal patterns of the chorus males in anurans are usually highly dynamic. Thus far, however, very few studies have addressed the network structure of vocal interactions in anurans.

The Emei music frog (*Babina daunchina*) is an excellent model system for investigating the dynamic patterns of vocal networks. Emei music frogs inhabit the edges of ponds covered with weeds during the breeding season, in southwest China (Ye, Fei, & Hu, 1993). Male frogs produce advertisement calls all day to attract females (Cui et al., 2011). Males call from either inside nests they have built or from outside the nests (Cui, Wang, Brauth, & Tang, 2010). Previous studies have shown that calls produced from inside nests are significantly more sexually attractive to females than those produced from outside because the burrow resonances modify the call spectrum in a way that is salient to both males and females (Cui, Tang, & Narins, 2012). Moreover, male advertisement calls consist of 3–8 notes, whose fundamental frequencies increase monotonically (Chen, Cui, Fang, Brauth, & Tang, 2011). Males can therefore modify their competitive efforts based on the perceived sexual attractiveness of rivals. Additionally, males are capable of interval timing and normally avoid producing advertisement calls that overlap the calls of other individuals (Fang et al., 2014). In view of these factors, it is logical to hypothesize that the vocal competitive patterns are influenced by conspecifics' vocalizations, leading to changes in network structure.

In the present study, we used SNA to quantify the dynamics of vocal network patterns in music frogs in the field. We focused on three network metrics that describe an individual's direct interactions in a group: strength, closeness centrality, and betweenness centrality (Croft et al., 2008). We chose these metrics because they measure different aspects of individual‐level position and permit us to understand group‐level structure (Farine & Whitehead, 2015; Wey et al., 2008). In the experiments, we simulated the vocalizations of unfamiliar intruders with different levels of competitiveness to examine whether network structure at the group level would change after the presence of a rival male. We predicted that male frogs would increase the rate of vocalizations, and therefore, the means of strength and closeness would increase, and the means betweenness would decrease when the competitiveness of the playback stimulus increased.

## 2 MATERIALS AND METHODS

### 2.1 Study site and subjects

Fieldwork was conducted in July and August 2017 in the Emei mountain area, Sichuan, China (29.36°N and 103.22°E, elevation of 941 m). Five ponds of various sizes (approximately 8.6 ± 3.1 m^2^) were selected as experimental populations in the present study, and each pond contained at least 3 male frogs. The distance between ponds ranged from 70 to 350 m, and frog calls carry approximately 50 m at most. Therefore, the distance between any two ponds was sufficiently great to prevent the activity of frogs in each pond from interfering with the behavior of frogs in any other pond during the performance of the experiments in the present study involving the presentation of acoustic stimuli.

Since female frogs show a preference for calls produce inside nests (inside calls; Cui et al., 2012) or calls with more note numbers (Cui et al., 2016), we broadcasted either inside calls or outside calls with three, five, or seven notes, respectively. Six categories of artificial stimuli were synthesized from natural calls used Adobe Audition 3.0 (Adobe) with 4 s intercall intervals (Cui et al., 2012; Fang et al., 2014). All stimuli were monophonic and equalized for intensity (75 dB SPL, re 20 μPa, measured at 1 m from the speaker).

We divided the experiment period into three equal 5 min time periods. In the first time period, we recorded the natural chorus using a digital voice recorder (Sony PCM‐D100) in order to determine the social network structure. Field playbacks were carried out in the second time period, during which one of six stimuli was played consecutively for 5 min to simulate an intruder. Since music frogs inhabit the edges of ponds, and call from either inside nests they have built or outside the nests (Cui et al., 2010), the playback was broadcasted from the edge of the pond and was located in a vacant position that outside the territory of adjacent frogs. We stopped broadcasting in the final time period and re‐recorded the natural chorus for another 5 min. To minimize the effects of previous playbacks, each pond was tested only once within the same day. We excluded the data if the number of calling males was less than 3 either in the first or the final time period and repeated the experiment on another day.

### 2.2 Social network

We identified individuals mainly based on location of males in the pond, and use their call characteristics for further confirmation. During our recording the natural chorus, we read the individual's ID (in a low voice) when a frog calling. A vocal interaction was defined as one frog's vocalization followed by another frog's vocalization. For each interaction, we recorded the start and the end time of calls and identities of the initiator and receiver. Dynamic and static vocal networks were constructed using the timeordered package (Blonder & Dornhaus, 2011). Three weighted network metrics were calculated for each individual in each network using the igraph package (Csardi & Nepusz, 2006). Strength: The sum of all edge weights connected to the focal individual (Farine & Whitehead, 2015), reflecting the frequency of social interactions. Closeness: The inverse of the shortest path length between the focal individual and every other individual (Freeman, 1979), which describes how well connected an individual is to all others in the network (Wey et al., 2008). Betweenness: A count of the number of shortest paths that flow through the focal individual (Farine & Whitehead, 2015), which describes how important a node is for social connections and stability of a social network. The removal of high‐betweenness individuals will likely fragment network connectivity (Wey et al., 2008). According to the definition of these metrics, strength and closeness would increase with the increased rate of vocalizations, whereas betweenness would decrease because the connection and stability of a social network had increased.

### 2.3 Statistical analyses

To estimate the consistency at the group level and to determine whether stronger rivals induce a higher level of vocal competition, we conducted generalized linear mixed models (GLMMs) with each network metric as the dependent variable. Note (3 notes, 5 notes, or 7 notes), location (calls from inside or outside nests), period (the first or the last time period), and their interaction were used as fixed effects. We included individual ID and pond as random effects.

Network metrics are inherently nonindependent and thus violate the assumptions underlying most parametric statistical tests. We determined significance in statistical tests (GLMMs) by comparing the coefficients of the models fitted to the observed data with coefficients calculated on 1,000 permutations of the network (Farine & Whitehead, 2015). Specifically, we performed 1,000 permutations using the timeordered package (Blonder & Dornhaus, 2011). Then, we calculated the same network metrics and conducted the same analysis for each permuted network. A result was considered significant if the coefficients of the models fitted to the observed data fell outside the 95% range of the permuted coefficient distribution (Leu, Farine, Wey, Sih, & Bull, 2016). GLMMs were analyzed using the lme4 package (Bates, Maechler, & Bolker, 2013). All statistical tests were performed using r 3.3.2 (R Core Team, 2016).

## 3 RESULTS

**Figure 1:**
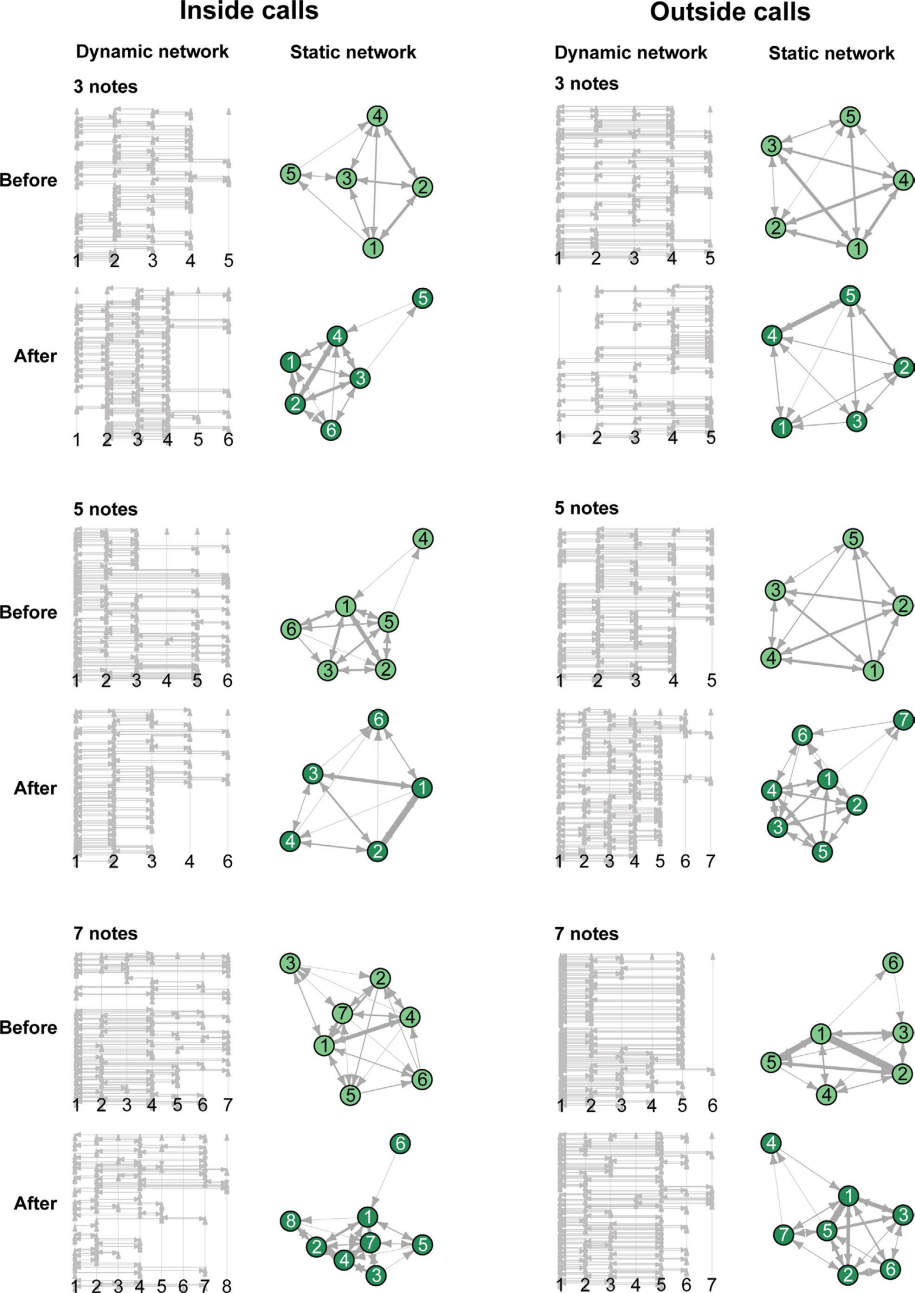
Dynamic networks static networks visualizations for all treatments for the first (before playbacks) and the third (after playbacks) time period. Dynamic networks: individuals (the numbers) move forward in time (vertical lines) and are connected by edges (horizontal lines) at different times, enabling simultaneous visualization of network topology and flow. Static networks: notes represent individuals and edges represent vocal interactions, the thickness of which reflects the strength of association. Arrows show directionality of vocal interactions. The visualizations of static networks were constructed using the Fuchterman–Reingold method in iGraph package (Csardi & Nepusz, 2006; Fruchterman & Reingold, 1991), which essentially pulls nodes that are highly connected closer together

**Figure 2:**
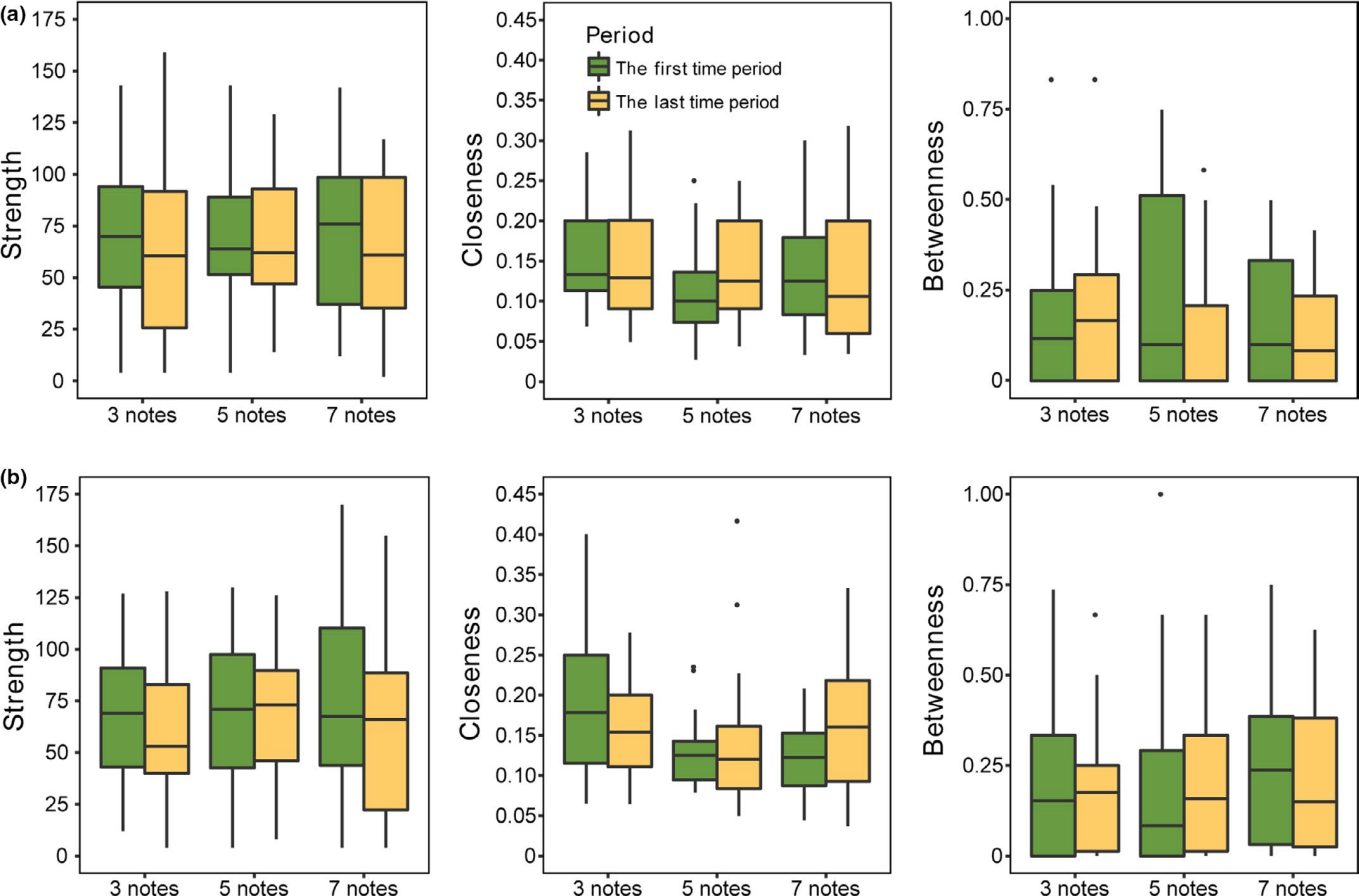
Three network metrics in the first and the last time period, (a) calls from inside nest, (b) calls from outside nest. Box plots show the median, interquartile ranges (IQR), 1.5 × IQR and outliers beyond this range (shown as points)

Each pond formed a well‐connected network during each observation period (Figure 1). We found that the members of a network or some individuals' position in a network changed between time period 1 and time period 2 (Figure 1). Nevertheless, the average strength (*t* = 0.236, *p* = .549), closeness (*t* = −1.761, *p* = .492), and betweenness (*t* = 1.540, *p* = .148) were consistent between these two periods in all ponds (Table 1, Figure 2, and Figure S1).

**Table 1:** GLMM (*p* values are in parentheses) coefficients for each network metric

Factor	Strength	Closeness	Betweenness
Period | before	1.787 (.549)	−0.030 (.492)	0.093 (.148)
Location | out	0.055 (.539)	0.004 (.342)	0.080 (.187)
Note | 7	−4.109 (.530)	−0.007 (.253)	0.002 (.199)
Note | 3	−9.084 (.504)	0.018 (.586)	0.072 (.188)
Period × location	2.457 (.424)	0.018 (.066)	−0.094 (.238)
Period × note | 7	6.948 (.484)	0.039 (.286)	−0.047 (.153)
Period × note | 3	6.270 (.481)	0.026 (.341)	−0.114 (.082)
Location × note | 7	2.720 (.478)	0.029 (.227)	0.001 (.417)
Location × note | 3	2.028 (.490)	0.001 (.457)	−0.096 (.518)
Period × location × note | 7	−1.242 (.466)	−0.067 (.457)	0.086 (.069)
Period × location × note | 3	−2.196 (.484)	0.016 (.055)	0.127 (.155)

Note

*p* Values were calculated by comparing the observed coefficient with 1,000 permuted coefficients. Factor reference categories were period | before: the first time period, location | out: out of nest, note | 7: 7 notes, note | 3: 3 notes.

We also found that neither location nor note had a significant effect on network metrics at the group level (all *p* > .05, Table 1, Figure 2, and Figure S1). In addition, period–location interactions, period–note interactions and period–location–note interactions had no significant effects on all 3 network metrics (all *p* > .05, Table 1 and Figure S1). In other words, there were no differences of the effects of the simulated intruder calls on the dynamics of the group‐level changes in network structure between simulated outside calls and inside calls, or as a function of different numbers of notes in the synthesized calls.

## 4 DISCUSSION

We simulated male intruders with different levels of sexual attractiveness in a natural population, and we used SNA to quantify changes in the dynamics of vocal interactions in Emei music frogs. We found that although some individuals' position in the network changed markedly after the disturbance (Figure 1), the mean strength, mean closeness, and mean betweenness of the network were consistent between two time periods (before and after introducing a simulated “intruder”), regardless of the intruder's competitiveness. This finding suggests that even though some individuals became more active and others became less active between the time periods, the frogs maintained the same mean strength of vocal interactions across time.

Some empirical studies have examined whether network metrics remain consistent over time at the individual or group level (Aplin et al., 2015; Blaszczyk, 2017; Frumkin et al., 2016). For example, a long‐term study on bighorn sheep (*Ovis canadensis*) demonstrated that individuals have consistent centrality in association networks across years (Vander Wal, Festa‐Bianchet, Reale, Coltman, & Pelletier, 2015). Similarly, a study showed that the network centrality of ringtailed lemurs (*Lemur catta*) can remain consistent between years, which provides evidence of social personalities (Kulahci et al., 2018). In the present study, we did not evaluate individual‐level repeatability directly, because some individuals stopped calling after the disturbance, even though they had been active during the first time period. In contrast, individuals that were silent in the first time period could join the chorus during the last time period (as shown in Figure 1). Therefore, we inferred that the individual's position in vocal network can change as a result of a conspecific disturbance.

An individual's position in a network may affect its fitness (Croft et al., 2008; Sih et al., 2009; Wey et al., 2008). For example, central individuals generally have higher social ranking (Bret et al., 2013), higher reproductive success (Bar Ziv et al., 2016; McDonald, 2007), and longer longevity (Lea, Blumstein, Wey, & Martin, 2010; Stanton & Mann, 2012). According to the definition of centralities, position in a network not only reflects an individual's relationship with all other individuals, but also the individual's ability to spread information throughout the network or control the information flow (Wey et al., 2008). In an anuran vocal network, individuals with higher strength or centrality generally vocalize more frequently, which may facilitate attracting mates. However, we found that some music frogs would stop calling to respond to simulated intruders with different competitiveness. The reason why individuals rarely vocalize with high intensity continuously is that they may avoid excess energy expenditure and increased predation risk. Another reason is that individuals may enhance their sexual attractiveness through increasing vocal complexity (Kelley, 2004; Zhu et al., 2017) or by choosing an optimal time for vocalizing (Byrne, 2008). For music frogs, males make flexible decisions about when to vocalize based on competitive context (Fang et al., 2014). Furthermore, males can modify the note numbers depending on a rival's vocalizations (Cui et al., 2016). Consequently, central individuals in a vocal network do not necessarily indicate a higher fitness.

We failed to find any significant interaction effects for period–location, period–note or period–location–note on network metrics, suggesting there is no difference among the effects of different treatments on group‐level changes in network structure. Study on fungus beetles (*Bolitotherus cornutus*) has shown that individual network position is repeatable in both the disturbed and undisturbed treatment groups, whereas disturbance has a significant effect on the consistency of overall network structure (Formica, Wood, Cook, & Brodie, 2017). In contrast, we found the vocal network structures at group level were consistent after different degree of disturbance. Our previous study has shown that male frogs preferentially compete with the highly sexually attractive calls (Jiang et al., 2015), which are produced from inside nests (Cui et al., 2012) or with calls containing relatively more notes (Cui et al., 2016). In the present study, the highest sexually attractive stimulation (inside calls with 7 notes) but not others induced some males to produce long calls that containing 10 notes in the last time period (observed data). In contrast, some other frogs reduced note numbers or even stopped calling in response to the same playback stimulation. These findings suggest that some frogs are more competitive in the presence of exotic rivals of high sexual attractiveness while other individuals tend to be less competitive under these circumstances. In addition, because the playback was closer to some individuals than to others, the closer males might respond more strongly to the simulated intruder. Therefore, the intensity of vocal communication at group level is relative stable may result from variation in how individuals respond to simulated competition varies substantially.

Although the vocal network that constructed in the present study had a relatively small size in spite of enough interactions within 5‐min sample period (approximately 81 times), and we only examined vocal networks at the group level and for relatively short time periods, it is clear from our observations that the structure of vocal networks in Emei music frogs remain stable at the group level after a conspecific disturbance, regardless of the intruder's competitiveness. Additionally, studies on the network structure of vocal interactions in anurans have been lacking, our study has implications for application of SNA on vocal communication in anurans. Future studies are required to investigate personality differences in Emei music frogs with an increased sample time, which so far remains largely unexplored (Kelleher, Silla, & Byrne, 2018). Such studies will advance our understanding of animal personality in amphibians and more importantly may also provide some insight to interindividual differences in vocal interactions.

## CONFLICT OF INTEREST

None declared.

## AUTHOR CONTRIBUTIONS

DK and CJG conceived the ideas and designed the experiment; DK collected field data, analyzed the data and drafted manuscript; CJG contributed to data collection and manuscript preparation.

## Data Availability

All data used in our analyses are available online at the Dryad Digital Repository: https://datadryad.org (https://doi.org/10.5061/dryad.55m7b3s).

## References

[ece35473-bib-0001] Aplin, L. M. , Firth, J. A. , Farine, D. R. , Voelkl, B. , Crates, R. A. , Culina, A. , … Sheldon, B. C. (2015). Consistent individual differences in the social phenotypes of wild great tits, *Parus major* . Animal Behaviour, 108, 117–127. 10.1016/j.anbehav.2015.07.016 26512142 PMC4579410

[ece35473-bib-0002] Bar Ziv, E. , Ilany, A. , Demartsev, V. , Barocas, A. , Geffen, E. , & Koren, L. (2016). Individual, social, and sexual niche traits affect copulation success in a polygynandrous mating system. Behavioral Ecology and Sociobiology, 70, 901–912. 10.1007/s00265-016-2112-4

[ece35473-bib-0003] Bates, D. , Maechler, M. , & Bolker, B. (2013). Linear mixed‐effect models using S4 classes. In: R package. Retrieved from http://CRAN.R-project.org/package=lme4

[ece35473-bib-0004] Blaszczyk, M. B. (2017). Consistency in social network position over changing environments in a seasonally breeding primate. Behavioral Ecology and Sociobiology, 72, 11.

[ece35473-bib-0005] Blonder, B. , & Dornhaus, A. (2011). Time‐ordered networks reveal limitations to information flow in ant colonies. PLoS ONE, 6, e20298 10.1371/journal.pone.0020298 21625450 PMC3098866

[ece35473-bib-0006] Botto, V. , & Castellano, S. (2016). Signal reliability and multivariate sexual selection in a lek‐breeding amphibian. Behavioral Ecology, 27, 1797–1807. 10.1093/beheco/arw115

[ece35473-bib-0007] Bret, C. , Sueur, C. , Ngoubangoye, B. , Verrier, D. , Deneubourg, J. L. , & Petit, O. (2013). Social structure of a semi‐free ranging group of mandrills (*Mandrillus sphinx*): A social network analysis. PLoS ONE, 8, e83015 10.1371/journal.pone.0083015 24340074 PMC3858359

[ece35473-bib-0008] Byrne, P. G. (2008). Strategic male calling behavior in an Australian Terrestrial Toadlet (*Pseudophryne bibronii*). Copeia, 2008(1), 57–63. 10.1643/CE-05-294

[ece35473-bib-0009] Chen, Q. , Cui, J. G. , Fang, G. Z. , Brauth, S. E. , & Tang, Y. Z. (2011). Acoustic analysis of the advertisement calls of the music frog, *Babina daunchina* . Journal of Herpetology, 45, 406–416. 10.1670/10-133.1

[ece35473-bib-0010] Croft, D. P. , Darden, S. K. , & Wey, T. W. (2016). Current directions in animal social networks. Current Opinion in Behavioral Sciences, 12, 52–58. 10.1016/j.cobeha.2016.09.001

[ece35473-bib-0011] Croft, D. P. , James, R. , & Krause, J. (2008). Exploring animal social networks. Princeton, NJ: Princeton University Press.

[ece35473-bib-0012] Csardi, G. , & Nepusz, T. (2006). The IGRAPH software package for complex network research. InterJournal Complex Systems, 1695, 1–9.

[ece35473-bib-0013] Cui, J. G. , Song, X. Y. , Fang, G. Z. , Xu, F. , Brauth, S. E. , & Tang, Y. Z. (2011). Circadian rhythm of calling behavior in the emei music frog (*Babina daunchina*) is associated with habitat temperature and relative humidity. Asian Herpetological Research, 2, 149–154.

[ece35473-bib-0014] Cui, J. G. , Song, X. W. , Zhu, B. C. , Fang, G. Z. , Tang, Y. Z. , & Ryan, M. J. (2016). Receiver discriminability drives the evolution of complex sexual signals by sexual selection. Evolution, 70, 922–927. 10.1111/evo.12889 26920078

[ece35473-bib-0015] Cui, J. G. , Tang, Y. Z. , & Narins, P. M. (2012). Real estate ads in Emei music frog vocalizations: Female preference for calls emanating from burrows. Biology Letters, 8, 337–340. 10.1098/rsbl.2011.1091 22158736 PMC3367746

[ece35473-bib-0016] Cui, J. G. , Wang, Y. S. , Brauth, S. , & Tang, Y. Z. (2010). A novel female call incites male‐female interaction and male‐male competition in the Emei music frog, *Babina daunchina* . Animal Behaviour, 80, 181–187. 10.1016/j.anbehav.2010.05.012

[ece35473-bib-0017] Deng, K. , Liu, W. , & Wang, D. (2017). Inter‐group associations in Mongolian gerbils: Quantitative evidence from social network analysis. Integrative Zoology, 12, 446–456. 10.1111/1749-4877.12272 28685954 PMC5725670

[ece35473-bib-0018] Fang, G. Z. , Jiang, F. , Yang, P. , Cui, J. G. , Brauth, S. E. , & Tang, Y. Z. (2014). Male vocal competition is dynamic and strongly affected by social contexts in music frogs. Animal Cognition, 17, 483–494. 10.1007/s10071-013-0680-5 24030652

[ece35473-bib-0019] Farine, D. R. , & Whitehead, H. (2015). Constructing, conducting and interpreting animal social network analysis. Journal of Animal Ecology, 84, 1144–1163. 10.1111/1365-2656.12418 26172345 PMC4973823

[ece35473-bib-0020] Fernandez, M. S. A. , Vignal, C. , & Soula, H. A. (2017). Impact of group size and social composition on group vocal activity and acoustic network in a social songbird. Animal Behaviour, 127, 163–178. 10.1016/j.anbehav.2017.03.013

[ece35473-bib-0021] Formica, V. , Wood, C. , Cook, P. , & Brodie, E. (2017). Consistency of animal social networks after disturbance. Behavioral Ecology, 28, 85–93. 10.1093/beheco/arw128

[ece35473-bib-0022] Freeman, L. C. (1979). Centrality in social networks conceptual clarification. Social Networks, 1, 215–239. 10.1016/0378-8733(78)90021-7

[ece35473-bib-0023] Fruchterman, T. M. J. , & Reingold, E. M. (1991). Graph drawing by force‐directed placement. Software‐practice & Experience, 21, 1129–1164. 10.1002/spe.4380211102

[ece35473-bib-0024] Frumkin, N. B. , Wey, T. W. , Exnicios, M. , Benham, C. , Hinton, M. G. , Lantz, S. , … Karubian, J. (2016). Inter‐annual patterns of aggression and pair bonding in captive American flamingos (*Phoenicopterus ruber*). Zoo Biology, 35, 111–119. 26882002 10.1002/zoo.21274

[ece35473-bib-0025] Jiang, F. , Fang, G. Z. , Xue, F. , Cui, J. G. , Brauth, S. E. , & Tang, Y. Z. (2015). Male music frogs compete vocally on the basis of temporal sequence rather than spatial cues of rival calls. Asian Herpetological Research, 6, 305–316.

[ece35473-bib-0026] Kelleher, S. R. , Silla, A. J. , & Byrne, P. G. (2018). Animal personality and behavioral syndromes in amphibians: A review of the evidence, experimental approaches, and implications for conservation. Behavioral Ecology and Sociobiology, 72, 79.

[ece35473-bib-0027] Kelley, D. B. (2004). Vocal communication in frogs. Current Opinion in Neurobiology, 14, 751–757. 10.1016/j.conb.2004.10.015 15582379

[ece35473-bib-0028] Kulahci, I. G. , Ghazanfar, A. A. , & Rubenstein, D. I. (2018). Consistent individual variation across interaction networks indicates social personalities in lemurs. Animal Behaviour, 136, 217–226. 10.1016/j.anbehav.2017.11.012

[ece35473-bib-0029] Kulahci, I. G. , Rubenstein, D. I. , & Ghazanfar, A. A. (2015). Lemurs groom‐at‐a‐distance through vocal networks. Animal Behaviour, 110, 179–186. 10.1016/j.anbehav.2015.09.016

[ece35473-bib-0030] Kurvers, R. H. J. M. , Krause, J. , Croft, D. P. , Wilson, A. D. M. , & Wolf, M. (2014). The evolutionary and ecological consequences of animal social networks: Emerging issues. Trends in Ecology & Evolution, 29, 326–335. 10.1016/j.tree.2014.04.002 24792356

[ece35473-bib-0031] Lea, A. J. , Blumstein, D. T. , Wey, T. W. , & Martin, J. G. A. (2010). Heritable victimization and the benefits of agonistic relationships. Proceedings of the National Academy of Sciences of the United States of America, 107, 21587–21592. 10.1073/pnas.1009882107 21115836 PMC3003113

[ece35473-bib-0032] Leu, S. T. , Farine, D. R. , Wey, T. W. , Sih, A. , & Bull, C. M. (2016). Environment modulates population social structure: Experimental evidence from replicated social networks of wild lizards. Animal Behaviour, 111, 23–31. 10.1016/j.anbehav.2015.10.001

[ece35473-bib-0033] Maldonado‐Chaparro, A. A. , Hubbard, L. , & Blumstein, D. T. (2015). Group size affects social relationships in yellow‐bellied marmots (*Marmota flaviventris*). Behavioral Ecology, 26, 909–915. 10.1093/beheco/arv034

[ece35473-bib-0034] McDonald, D. B. (2007). Predicting fate from early connectivity in a social network. Proceedings of the National Academy of Sciences of the United States of America, 104, 10910–10914. 10.1073/pnas.0701159104 17576933 PMC1904119

[ece35473-bib-0035] Montiglio, P. O. , McGlothlin, J. W. , & Farine, D. R. (2018). Social structure modulates the evolutionary consequences of social plasticity: A social network perspective on interacting phenotypes. Ecology and Evolution, 8, 1451–1464. 10.1002/ece3.3753 29435224 PMC5792542

[ece35473-bib-0036] Patriquin, K. J. , Leonard, M. L. , Broders, H. G. , & Garroway, C. J. (2010). Do social networks of female northern long‐eared bats vary with reproductive period and age? Behavioral Ecology and Sociobiology, 64, 899–913. 10.1007/s00265-010-0905-4

[ece35473-bib-0037] Pinter‐Wollman, N. , Hobson, E. A. , Smith, J. E. , Edelman, A. J. , Shizuka, D. , de Silva, S. , … McDonald, D. B. (2014). The dynamics of animal social networks: Analytical, conceptual, and theoretical advances. Behavioral Ecology, 25, 242–255. 10.1093/beheco/art047

[ece35473-bib-0038] R Core Team (2016). R: A language and environment for statistical computing. Vienna, Austria: R Foundation for Statistical Computing Retrieved from www.r-project.org

[ece35473-bib-0039] Sih, A. , Hanser, S. F. , & McHugh, K. A. (2009). Social network theory: New insights and issues for behavioral ecologists. Behavioral Ecology and Sociobiology, 63, 975–988. 10.1007/s00265-009-0725-6

[ece35473-bib-0040] Snijders, L. , & Naguib, M. (2017). Communication in animal social networks: A missing link? Advances in the Study of Behavior, 49, 297–359.

[ece35473-bib-0041] Stanton, M. A. , & Mann, J. (2012). Early social networks predict survival in wild bottlenose dolphins. PLoS ONE, 7, e47508 10.1371/journal.pone.0047508 23077627 PMC3471847

[ece35473-bib-0042] VanderWaal, K. L. , Atwill, E. R. , Isbell, L. A. , & McCowan, B. (2014). Linking social and pathogen transmission networks using microbial genetics in giraffe (*Giraffa camelopardalis*). Journal of Animal Ecology, 83, 406–414. 24117416 10.1111/1365-2656.12137

[ece35473-bib-0043] Vander Wal, E. , Festa‐Bianchet, M. , Reale, D. , Coltman, D. W. , & Pelletier, F. (2015). Sex‐based differences in the adaptive value of social behavior contrasted against morphology and environment. Ecology, 96, 631–641. 10.1890/14-1320.1 26236860

[ece35473-bib-0044] Wells, K. D. , & Schwartz, J. J. (2007). The behavioral ecology of anuran communication In Narins P. M., Feng A. S., Fay R. R., & Popper A. N. (Eds.), Hearing and sound communication in amphibians (pp. 44–86). New York, NY: Springer Verlag.

[ece35473-bib-0045] Wey, T. , Blumstein, D. T. , Shen, W. , & Jordán, F. (2008). Social network analysis of animal behaviour: A promising tool for the study of sociality. Animal Behaviour, 75, 333–344. 10.1016/j.anbehav.2007.06.020

[ece35473-bib-0046] Wey, T. W. , Burger, J. R. , Ebensperger, L. A. , & Hayes, L. D. (2013). Reproductive correlates of social network variation in plurally breeding degus (*Octodon degus*). Animal Behaviour, 85, 1407–1414. 10.1016/j.anbehav.2013.03.035 24511149 PMC3914217

[ece35473-bib-0047] Xu, F. , Cui, J. G. , Song, J. , Brauth, S. E. , & Tang, Y. Z. (2012). Male competition strategies change when information concerning female receptivity is available. Behavioral Ecology, 23, 307–312. 10.1093/beheco/arr187

[ece35473-bib-0048] Ye, C. Y. , Fei, L. , & Hu, S. Q. (1993). Rare and economic amphibians of China. Chengdu, China: Sichuan Publishers House of Science & Technology.

[ece35473-bib-0049] Zhu, B. , Wang, J. , Sun, Z. , Yang, Y. , Wang, T. , Brauth, S. E. , … Cui, J. (2017). Competitive pressures affect sexual signal complexity in *Kurixalus odontotarsus*: Insights into the evolution of compound calls. Biology Open, 6, 1913–1918. 29175862 10.1242/bio.028928 PMC5769655

