# Supplementary figures and images for: Vocal networks remain stable after a disturbance in Emei music frogs
Source: Ecol Evol. 2019 Jul 23;9(16):9290–7. doi: 10.1002/ece3.5473 (PMC6706236; doi:10.1002/ece3.5473)

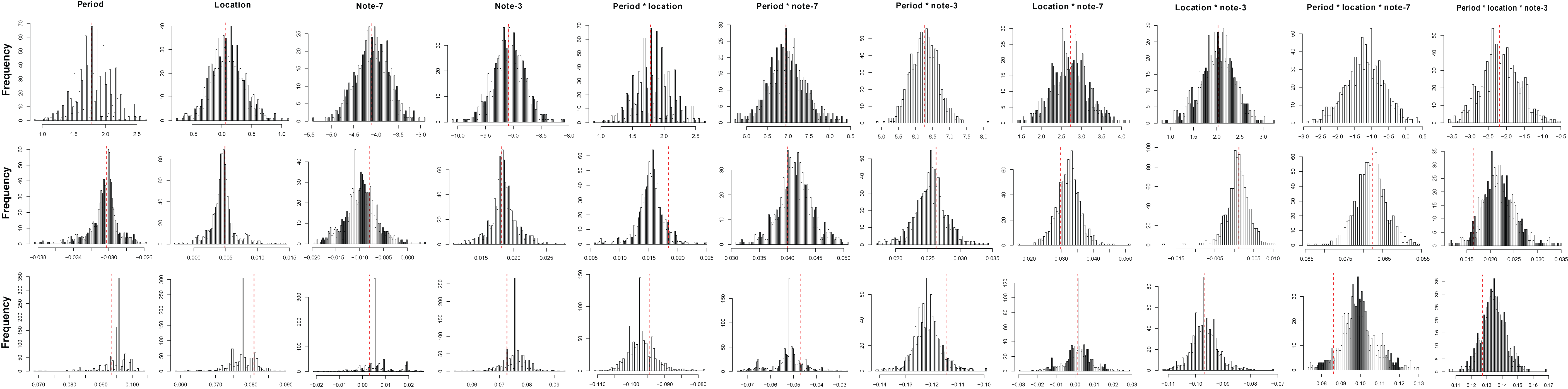

Supplement: Supplementary file 1 [file ECE3-9-9290-s001.tif]
